# Fatal Cobalt Toxicity after a Non-Metal-on-Metal Total Hip Arthroplasty

**DOI:** 10.1155/2017/9123684

**Published:** 2017-08-27

**Authors:** Rinne M. Peters, Pax Willemse, Paul C. Rijk, Mels Hoogendoorn, Wierd P. Zijlstra

**Affiliations:** ^1^Department of Orthopedic Surgery, Medical Center Leeuwarden, Henri Dunantweg 2, 8934 AD Leeuwarden, Netherlands; ^2^Department of Internal Medicine, Medical Center Leeuwarden, Henri Dunantweg 2, 8934 AD Leeuwarden, Netherlands

## Abstract

This case illustrates the potential for systemic cobalt toxicity in non-metal-on-metal bearings and its potentially devastating consequences. We present a 71-year-old male with grinding sensations in his right hip following ceramic-on-ceramic total hip arthroplasty (THA). After diagnosing a fractured ceramic liner, the hip prosthesis was revised into a metal-on-polyethylene bearing. At one year postoperatively, X-rays and MARS-MRI showed a fixed reversed hybrid THA, with periarticular densities, flattening of the femoral head component, and a pattern of periarticular metal wear debris and pseudotumor formation. Before revision could take place, the patient was admitted with the clinical picture of systemic cobalt toxicity, supported by excessively high serum cobalt and chromium levels, and ultimately died. At autopsy dilated cardiomyopathy as cause of death was hypothesized. A third body wear reaction between ceramic remnants and the metal femoral head very likely led to excessive metal wear, which contributed systemic cobalt toxicity leading to neurotoxicity and heart failure. This case emphasizes that fractured ceramic-on-ceramic bearings should be revised to ceramic-on-ceramic or ceramic-on-polyethylene bearings, but not to metal-on-polyethylene bearings. We aim to increase awareness among orthopedic surgeons for clinical clues for systemic cobalt intoxication, even when there is no metal-on-metal bearing surface.

## 1. Introduction

The development of systemic cobalt toxicity after total hip arthroplasty (THA) appears to be very rare [1, 2]. Case reports have highlighted systemic cobalt toxicity after failed metal-on-metal (MoM) articulations [1, 3, 4]. Therefore, most practicing orthopedic surgeons probably associate systemic cobalt toxicity only with MoM bearings. However, revision of a broken ceramic liner to a MoP articulation can also cause mechanical wear and subsequent release of cobalt ions, due to contact between the new cobalt-chromium (CoCr) femoral head and remaining ceramic particles, which may become embedded in the PE inlay [1, 2, 5–9]. Local consequences of this third body wear process include metallosis and hip complaints and, in more severe cases, migration into periarticular soft tissues and subsequent systemic repercussions such as cobalt toxicity (hypothyroidism, polycythemia, cardiomyopathy, cognitive dysfunction, neuropathy, and fatigue). We present a rare case of suspected systemic cobalt toxicity following revision of a failed CoC THA with fatal outcome.

## 2. Case Report

A 71-year-old male with a history of diabetes and multiple myeloma visited the orthopedic outpatient clinic of a level 2 trauma hospital in 2004, with clinical and radiological signs of end-stage osteoarthritis of the right hip. The decision to proceed with total hip arthroplasty (THA) was made. A cementless ABG type II (Anatomique Benoist Gerard; Stryker Howmedica Osteonics, Newbury, UK) with a ceramic-on-ceramic (CoC) articulation was implanted. The postoperative course was uncomplicated.

**Figure 1:**
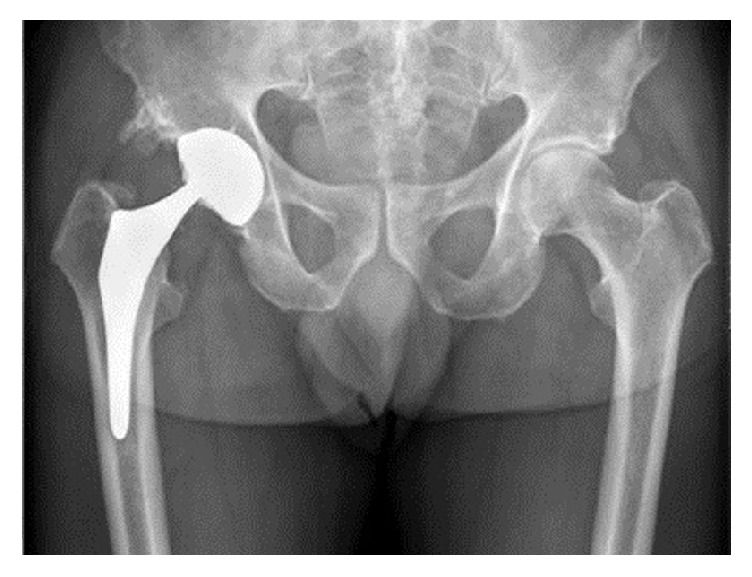
AP radiograph demonstrating eccentric positioning of the femoral head component of the right total hip arthroplasty without evidence for subsidence of the stem and loosening of the components.

In 2014, the patient perceived a squeaking sound and experienced increasing pain on the lateral side of the right hip after physical exercise. The patient denied prior trauma to the hip. The pain was present during flexion of the hip and he was not able to bear weight on his right leg. Clinical examination revealed pain on the lateral side of the hip upon flexion and inguinal pain during rotation. Alternative axial loading and traction to the hip joint were painful (positive push-pull test). Plain radiographs demonstrated a normal configuration of the acetabular component, albeit with an eccentric positioning of the head within the socket; there was no evidence for osteolytic lesions, subsidence of the stem, or loosening of the components (Figure 1).

A skeletal scintigraphy excluded aseptic loosening of the prosthesis and no increased intensity around the femoral and acetabular components was observed. No signs of infection were present in whole blood samples. Considering the typical complaints and findings on clinical examination a fracture of the ceramic liner was suspected, and a revision procedure was performed.

**Figure 2:**
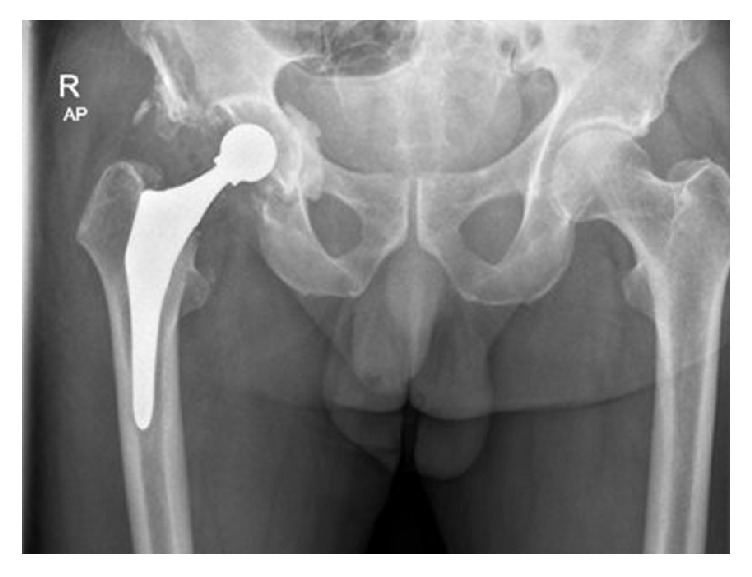
AP radiograph, 6 weeks following revision arthroplasty, demonstrating a polyethylene cup combined with a cobalt-chromium (CoCr) femoral head.

Intraoperatively, more than 20 ceramic pieces of the broken liner were detected and thoroughly removed and the surgical wound was repeatedly flushed. Macroscopically, all ceramic particles were removed. The joint capsule demonstrated hypertrophic changes for which synovectomy and debridement were performed. The femoral stem and acetabular shell did not show any signs of loosening. The acetabular shell was removed and replaced by a polyethylene (PE) SHP cup (Scientific Hip Prosthesis; Biomet, Warsaw, Indiana, USA) combined with a 28 mm cobalt-chromium (CoCr) alloy femoral head, with a matching taper (Figure 2).

**Figure 3:**
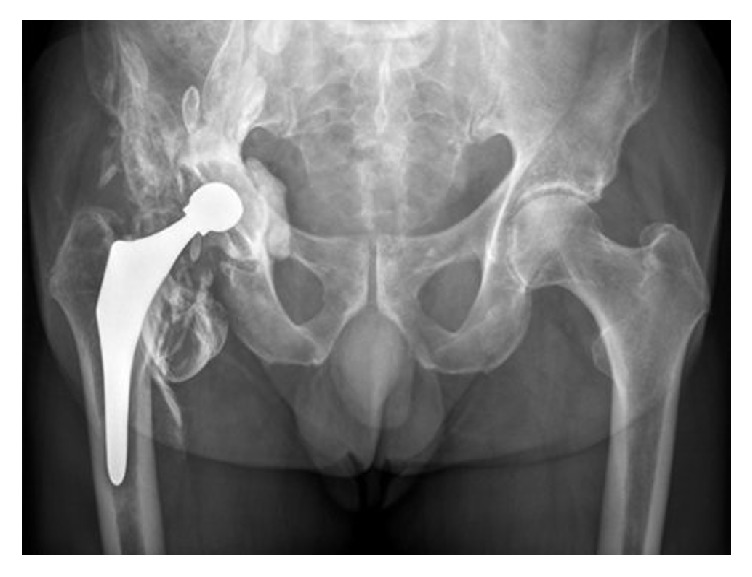
AP radiograph, one year postoperative. Periarticular densities around all components with flattening of the femoral head.

**Figure 4:**
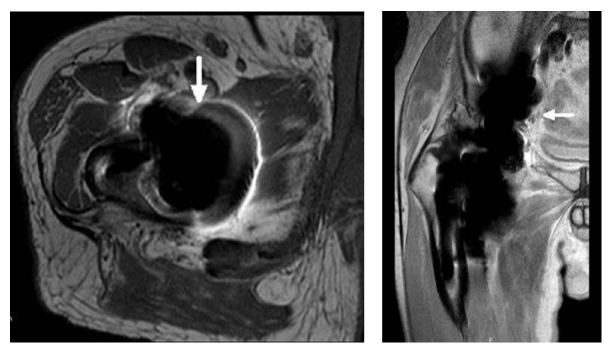
MARS-MRI of the right hip with metal wear debris with comprehensive pseudotumor formation (arrow).

Postoperatively, he recovered well. At 6 weeks follow-up, the patient perceived an excellent function of the hip and there were no signs of radiological abnormalities. Six months after the revision procedure, the patient developed more pain in the right hip. To exclude progression of his multiple myeloma, a fluorine-18-fluorodeoxyglucose-positron emission tomography with computer tomography was performed, which showed no active osteolytic lesions but revealed metabolic activity in the right hip and gluteus muscle. Additional plain radiographs showed a well-fixed reversed hybrid total hip prosthesis and impressive periarticular densities around the components combined with flattening of the femoral head component with loss of its sphericity (Figure 3). Therefore, a magnetic resonance image (MRI) with metal artefact reduction sequence (MARS) was obtained which demonstrated metal wear debris with comprehensive pseudotumor formation surrounding the components of the total hip prosthesis (Figure 4). A second revision procedure was proposed to the patient but had to be postponed due to an ischemic cerebrovascular accident, for which both acetylsalicylic acid and dipyridamole were initiated. Hypothyroidism was diagnosed for which thyroxine supplementation was prescribed.

Two weeks later, the patient was admitted in our hospital due to overall degradation of health with abdominal pain, vomiting, and diarrhea for which he received broad-spectrum antibiotics, hydrocortisone, and bicarbonate. Furthermore, the patient reported asymmetrical hearing loss, visual impairment, complaints of vertigo, and unintentional weight loss. Within 2 days, the patient deteriorated and subsequently died. At autopsy, a hypertrophic heart with a weight of 615 gram (*n*: 350–400 g) without any coronary artery disease was observed. No signs of stenosis, thrombi, or dissection of the coronary arteries were found. Serum metal ion analysis, obtained 13 days before death but only available a view days before death, showed excessively high cobalt (596,5 *μ*g/l; normal range: 0–2,4 *μ*g/l) and chromium levels (48,8 *μ*g/l; normal range 0–2,1 *μ*g/l) [10]. It was hypothesized that due to third body wear reaction between ceramic remnants and the metal femoral head, severe and ultimately fatal cobalt toxicity occurred with dilated cardiomyopathy and neurotoxicity (hearing loss and visual impairment). The authors have obtained written informed consent from all subjects involved in this case for print and electronic publication of the case report.

## 3. Discussion

We demonstrate a very rare case of systemic cobalt toxicity following revision of a failed CoC THA with fatal outcome. The aim of this case report is to emphasize the potential for systemic cobalt toxicity after revision of a broken ceramic liner into a metal-on-polyethylene (MoP) bearing, and its potentially devastating consequences. Our report provides comprehensive imaging including extensive metallosis, periarticular metal artefacts, flattening of the femoral head, and pseudotumor formation. To the best of our knowledge, this is the second reported case of suspected fatal cobalt toxicity secondary to catastrophic wear of a metal-on-polyethylene bearing following revision of a fractured ceramic component. In addition, MRI of the affected hip joint of in these patients has not been published before.

Local and systemic repercussions of cobalt toxicity have been described after THA from various bearing types. Systemic cobalt toxicity after failed metal-on-metal (MoM) articulations [1, 3, 4] as well as following failed CoC THA and subsequent revision to a MoP or MoM articular pairing was observed by several authors [1, 2, 5–9]. CoC bearings in THA were introduced as alternative bearing type in young, active patients because of low rates of wear and minimal reactivity to wear debris [11, 12]. Since the introduction in the 1970s, ceramic articulations have undergone several changes aiming to improve fixation, wear characteristics, and resistance to component fractures [2, 5, 13, 14]. The incidence of fractured ceramic components is unknown. Manufacturer retrieval data demonstrate a decreased incidence of femoral head fractures as the production quality improved from 13,4% for components manufactured before 1990 to 0.004% for ceramic Biolox® femoral heads produced after 1994 [15]. However, clinical studies demonstrate fracture rates varying from 0,5% (40 out of 8,022 CoC THAs) [16] to 1.1% [17]. Contributing factors responsible for fractures are inappropriate geometry of the head/neck design, iatrogenic damage during surgery, and in case of acetabular component fractures, by wrong socket positioning, implantation at inappropriate angles [14].

### 3.1. Revision

Treatment of a failed CoC THA in patients with well-fixed components is challenging and information on the long-term treatment results is scarce [2, 14]. After a fracture of a ceramic component, both surgeon and patients may be reluctant to implant another CoC articulation given concerns about a refracture. In order to eliminate the risk of a refracture after revision, surgeons and patients may opt for revision to a MoP articulation. Good long-term results of such revision have been described [18].

Ideally, all fractured ceramic particles should be removed during revision surgery. Based on the size of the ceramic particles (5 *μ*m in diameter), intraoperative localization and elimination of all particles seem to be not feasible. To remove all fragments of a fractured ceramic implant, the principles of tumor surgery should probably be followed, aiming for macroscopically clear margins with gross synovectomy. Nonetheless, even then microscopic particles may still be present locally. Since it is impractical to enforce this requirement in revision surgery, surgeons have to perform thorough lavage of the soft tissue and extensive synovectomy to remove as much of the ceramic particles as possible. However, it must be expected that ceramic particles will remain in adjacent tissue and may become embedded in a PE inlay, causing abrasive wear of the relatively softer CoCr femoral head [5, 9]. Subsequently, nanoparticles (third body wear process) from the metal head are released, which undergo corrosion, resulting in the release of cobalt and chromium ions. This could potentially lead to metallosis, subsequent migration of metal degradation products into pericapsular soft tissues, and, in more severe cases, systemic repercussions such as cobalt toxicity [2, 5–8]. On radiographs, loss of the spherical profile of the femoral head component and periarticular densities might be encountered [6, 9].

### 3.2. Cobalt Toxicity

Historically, cobalt toxicity became a recognized clinical problem after recreational consumption of beer with a foam-stabilizing agent containing cobalt sulphate or cobalt chloride. The addition of cobalt to beer was considered to play a key role in the development of the low-output ‘beer-drinkers' cardiomyopathy, first described in Quebec, Canada, in the nineteen sixties [1, 19]. The clinical presentation during that epidemic was quite similar to the case presented here, with rapidly progressive dysfunction of several vital organs in otherwise fairly healthy persons. Cobalt toxicity also has been described after industrial exposure to cobalt, as well as iatrogenic toxicity following treatment of anaemia with cobalt-chloride tablets [1, 2]. In more recent years, several cases of cobalt toxicity have been related to cobalt containing implants used in joint arthroplasty, especially in those with overt prosthetic failure or resurfacing of the MoM THA [1]. However, a narrative review on systemic cobalt toxicity after THA revealed that 8 of the 18 reported cases of cobalt toxicity were encountered in patients with non-metal-on-metal articulations [7].

Cobalt is a trace metal element which is essential for normal cellular metabolism but at high levels may lead to reduced human osteoblast activity, changes in osteoprotegerin (OPG)/receptor activator of nuclear factor kappa B ligand (RANKL) ratio leading to oxidative DNA damage, cellular apoptosis, necrosis, and oxidative DNA damage [1, 20]. Subsequently, elevated cobalt levels can elicit a multitude of symptoms including cardiomyopathy, hypothyroidism, polycythemia, cognitive dysfunction, neuropathy, and fatigue [1, 21].

This case report should be considered in the light of having certain limitations. First of all, no histological samples of the hip were obtained at autopsy. In addition, no samples of the myocardium were tested for elevated cobalt levels or mitochondrial damage. Mitochondrial injury by cobalt or other trace elements leading to low-output cardiomyopathy has been reported in literature [1]. Lastly, cobalt toxicity as cause of death in this case cannot be absolutely confirmed, because significant preexisting comorbidities such as multiple myeloma and diabetes might have contributed to his death. However, several features support fatal cobalt toxicity. These include very high levels of serum cobalt, measured short before dying, in association with observed cardiomyopathy without significant coronary artery disease at autopsy, the rapid deterioration of several neurologic functions, the recently diagnosed hypothyroidism, and the visual symptoms. All the above cardiac and noncardiac organ involvement has been described in association with systemic cobalt toxicity [1]. Furthermore, the patient had no other sources of hard metal contamination apart from the THA, and the excessively elevated serum cobalt levels were measured simultaneously with the radiological appearance of metal artefacts on plain radiographs of the right hip.

## 4. Conclusion

We aim to highlight the potential for systemic symptoms of cobalt toxicity in patients without a MoM THA. Revision surgeons should recognize the clinical clues for systemic cobalt intoxication such as hypothyroidism, polycythemia, cardiomyopathy, cognitive dysfunction, neuropathy, and fatigue, even when there is no MoM bearing surface. We recommend that a failed CoC articulation should be revised to CoC or ceramic-on-polyethylene (CoP) bearings. Since ceramic particles may become embedded into the articular surface after revision, causing third body wear, relatively softer MoP or MoM bearings should be avoided [2, 21, 22]. Additionally, the surgeon must assure that contributing factors that increase the risk of a new ceramic fracture should be addressed during revision surgery. In case of revision of a failed CoC THA to a THA with a metal femoral head component, serial cobalt ion monitoring and radiographic screening are strongly advised.
